# Protein Beverage *vs.* Protein Gel on Appetite Control and Subsequent Food Intake in Healthy Adults

**DOI:** 10.3390/nu7105421

**Published:** 2015-10-21

**Authors:** Sha Zhang, Heather J. Leidy, Bongkosh Vardhanabhuti

**Affiliations:** 1 Food Science Program, Division of Food Systems and Bioengineering, University of Missouri, Columbia, MO 65211, USA; zhangsha1218@hotmail.com; 2 Nutrition and Exercise Physiology, School of Medicine; University of Missouri, Columbia, MO 65211, USA; leidyh@health.missouri.edu

**Keywords:** whey protein, satiety, beverage, gel, food intake

## Abstract

The objective of this study was to compare the effects of food form and physicochemical properties of protein snacks on appetite and subsequent food intake in healthy adults. Twelve healthy subjects received a standardized breakfast and then 2.5 h post-breakfast consumed the following snacks, in randomized order: 0 kcal water (CON) or 96 kcal whey protein snacks as beverages with a pH of either 3.0 (Bev-3.0) or 7.0 (Bev-7.0) or gels as acid (Gel-Acid) or heated (Gel-Heated). *In-vitro* study showed that Bev-3.0 was more resistant to digestion than Bev-7.0, while Gel-Acid and Gel-Heated had similar digestion pattern. Appetite questionnaires were completed every 20 min until an *ad libitum* lunch was provided. Post-snack hunger, desire to eat, and prospective food consumption were lower following the beverages and gels *vs.* CON (all, *p* < 0.05), and post-snack fullness was greater following the snacks (except for the Bev-3.0) *vs.* CON (all, *p* < 0.05). Gel-Heated treatment led to lower prospective food consumption *vs.* Bev-3.0; however, no other differences were detected. Although all snacks reduced energy intake *vs.* CON, no differences were observed among treatments. This study suggested that whey protein in either liquid or solid form improves appetite, but the physicochemical property of protein has a minimal effect.

## 1. Introduction

There is growing evidence illustrating that increased dietary protein increases weight loss and prevents weight gain, potentially by promoting satiety [1,2]. Among all the different sources of protein, whey protein has been suggested to be superior to other proteins in promoting satiety [3,4,5]. The mechanism has been proposed to be due to its fast digestion behavior, unique amino acid profile, and stimulation of satiety hormones in the gastrointestinal system [1,4,6,7]. However, there are several factors that may alter the beneficial effects of whey protein, including timing of protein consumption, protein quantity and physical form.

Food in liquid form is generally considered to elicit weaker appetite response compared to food in solid form [8]. There is accumulating evidence suggested that liquid carbohydrates commonly produce less satiety than solid ones [9,10]. With respect to whether food form plays a role when consuming protein-rich foods, several studies illustrate reductions in appetite and increases in daily food intake with the consumption of high protein beverages compared to high protein meals [11,12]. However, it is important to note that protein in a liquid system can vary in structure and physical properties depending on their target functional properties. Likewise, protein gels can be formed with different structure and physicochemical properties depending on protein concentration, pH, and heating conditions [13,14,15]. Differences in the physical form and physicochemical properties of proteins could likely lead to differences in their gastric behavior and thus affect satiety property. Studies using *in vitro* gastric simulation showed that whey protein liquid and/or gel with different structural characteristics changed digestion behavior [16,17,18,19]. Whey proteins subjected to different thermal treatments result in protein aggregates with different aggregate size, viscosity, surface hydrophobicity, and solubility, which further determine its susceptibility to hydrolysis [20]. Whey protein liquid heated at neutral pH is found to be more susceptible to proteolysis than that formed at acidic pH [21]. Whey protein gels formed close to the isoelectric point are more resistant to simulated gastric digestion than gels formed at neutral pH [22]. Although evidence demonstrates that whey protein in different mediums and with different structures exhibits different *in vitro* gastric behavior, to the best of our knowledge, no study has used clinical trials to test whether such differences exist *in vivo*.

The purpose of this study was to assess whether the consumption of mid-morning whey protein snacks in different physical forms (beverage *vs.* gel) and with different structure, could influence appetite and subsequent food intake.

## 2. Methods

### 2.1. Clinical Trial

#### 2.1.1. Participants

The difference between treatments in postprandial hunger and fullness from our previous study [23] that compared a 0 kcal pre-load *vs.* 5 g protein pre-load (effect sizes: 0.92 and 1.09, respectively) indicated that a sample size of *n* = 9–12 would lead to 80% power to detect differences in these outcomes in the current study. Thus, a sample size of *n* = 12 was incorporated. Twelve participants, aged 18–30 years, were recruited at the University of Missouri Columbia campus using advertisements and flyers. Eligibility included the following: (1) normal to overweight (BMI: 18–28 kg/m^2^); no metabolic, psychological, or neurological diseases/conditions; (2) not having been clinically diagnosed with an eating disorder; (3) not currently/previously on a weight loss or other special diet (in the past six months); (4) not a smoker (in the past year); (5) habitually eat breakfast between 6:00–9:00 AM and lunch between 11:00 AM–2:00 PM; (6) no food allergies or intolerances to dairy products; (7) not pregnant; (8) not taking any medications, or have had no changes in medication within the past six months, that could influence the study outcomes. To minimize variability, all participants were asked to have a standardized breakfast at the same time of day and to refrain from drinking alcohol and undertaking prolonged vigorous physical activity the day before the test visit. The study protocol was approved by the Institutional Review Board (IRB) at the University of Missouri, and all participants were provided with informed consent. The participants received $150 for completing all study procedures.

#### 2.1.2. Study Design

The study followed a randomized-crossover design to compare the consumption of mid-morning whey protein snacks in liquid and solid form. Each participant was asked to complete 5, 3-h testing days. On each testing day, the participants consumed a standardized breakfast at home, and then 2.5 h after breakfast, they were asked to report to our facility to complete the testing day. Standardized breakfast included Smart Ones Morning Express Breakfast Quesadilla (230 kcal, 12 g protein, 29 g carbohydrates and 7 g fat per one quesadilla) and Dole Pinneapple Tidmits in 100% juice (60 kcal, 1 g protein, 15 g carbohydrates, and 0 g fat per one cup).

Each participant was asked to consume one of the following mid-morning snacks: 0 kcal water (CON) or ~100 kcal whey protein snacks (24 g protein) as beverages with a pH of either 3.0 (Bev-3.0) or 7.0 (Bev-7.0) or gels as acid (Gel-Acid) or heated (Gel-Heated). Perceived appetite questionnaires were completed before and every 20 min after the snack was consumed. At 2 h after the mid-morning snack, the participants were provided with an *ad libitum* lunch. Participants were given Lean Pockets Chicken Parmesan (270 kcal, 10 g protein, 40 g carbohydrates and 7 g fat per one pocket). The pockets were cut into 4 sections, and the participants were provided with 4 sections on one plate with additional pocket pieces provided as needed. The participants consumed as much or as little of the lunch as they desired until feeling “comfortably full” over a 20 min period. Energy intake from the lunch was calculated according to the weight of the meal.

#### 2.1.3. Protein Snacks

The dietary characteristics of the snacks are shown in Table 1. The Bev-3.0 and Bev-7.0 were prepared by dissolving whey protein isolate powder (Davisco Foods International, Le Sueur, MN, USA) in water at 8% (g/g), adding sweetener and flavor, adjusting pH to the desired values, and then heating at 85 °C for 30 min. For the Gel-Acid, glucono-δ-lactone (GDL) was added to whey protein beverage at pH 7.0 to reach a final pH of 5.0 after 24 h incubation. The Gel-Heated was prepared by heating 12% protein at pH 7.0 with sweetener and flavor at 85 °C for 30 min. All treatments, except CON, had 24 g protein per serving. The serving size of Bev-3.0, Bev-7.0, and Gel-Acid was 300 g. To control the total amount of the serving size, 200 g of Gel-heated sample was served with 100 g water.

**Table 1:** Dietary characteristics of each snack.

	Bev-3.0	Bev-7.0	Gel-Heated	Gel-Acid	CON
Energy content (kcal)	96	96	96	96	0
Total protein (g)	24	24	24	24	0
Protein content (%)	8	8	12	8	0
pH	3.0	7.0	7.0	5.0	7.0
Serving size *	300 g	300 g	200 g	300 g	300 g
Food type	Beverage	Beverage	Gel	Gel	Beverage
Flavor	Raspberry	Vanilla	Vanilla	Vanilla	-
Sweetener	Sucralose	Sucralose	Sucralose	Sucralose	-

* 100 g water was also served such that the total weight of the meal consumed was 300 g. Snacks: 0 kcal water (CON) or 96 kcal whey protein snacks as beverages with a pH of either 3.0 (Bev-3.0) or 7.0 (Bev-7.0) or gels as acid (Gel-Acid) or heated (Gel-Heated).

#### 2.1.4. Meals

The standardized breakfast consumed on the morning of all testing days consisted of breakfast quesadillas and pineapple cup. Each participant was given the option of consuming 1 or 2 breakfast quesadillas depending on the person’s individual needs but were required to consume the pineapple cup. Thus, the mean energy content of the breakfast was 380.38 ± 104.67 kcal. Participants consumed the breakfast at home with the same amount at the same time between 6 A.M.–9 A.M. for each testing day. They were also instructed to return all wrappers associated with the pack-put breakfast as well as any potential remains. The *ad libitum* lunch consisted of bite-size chicken parmesan pizza pockets and water. The energy and macronutrient content of the pockets/serving were 270 kcal; 10 g protein/10 g carbohydrates/7 g fat. The participants were instructed to eat as much or as little until feeling comfortably full within 20 min. Additional pizza pocket pieces were given to the participants as needed. All contents were pre-weighed at the time of serving and the remaining contents were weighed after the meal to determine the amount consumed. Total energy intake was then calculated according to the weight.

#### 2.1.5. Appetite Questionnaire

The participants were instructed to complete the questionnaire every 20 min throughout each testing. The questionnaire assessed perceived sensations of hunger, fullness, desire to eat, prospective food consumption, and palatability (*i.e.*, overall liking). The questionnaire contained validated visual analog scales (VAS) incorporating a 100 mm horizontal line rating scale for each response [24]. The scales were anchored with labels 0 = no at all and 100 = extremely.

#### 2.1.6. Data and Statistical Analysis

Net incremental area under the curve (AUC) was calculated from the pre and post-snack time points for hunger, fullness, desire to eat, and prospective food consumption [25]. The dietary compensation of the protein snack (96 kcal) was added to the subsequent energy intake. A repeated measures ANOVA was applied to compare the main effects of snacking on appetite and subsequent energy intake. When main effects were detected (*p* < 0.05), post-hoc pair wise comparisons were performed using Least Significant Differences to identify differences between snacks.

Pearson correlational analyses were performed to identify the relationship between sensory attributes of the snack and study outcomes. Overall liking was found to be significantly associated with hunger (*r*. 0.386, *p* < 0.05), fullness (*r*. 0.422, *p* < 0.05), desire to eat (*r*. 0.353, *p* < 0.05), and prospective food consumption (*r*. 0.335, *p* < 0.05), thus it was included as a potential covariate using a mixed factor ANOVA. Because it did not act as a covariate on all of the outcomes, the data is reported as unadjusted means. Data analyses were conducted using the Statistical Package for the Social Sciences (SPSS; version 22.0; Chicago, IL, USA). *p* < 0.05 was considered statistically significant.

### 2.2. In Vitro Digestion

Protein snacks for *in vitro* digestion were prepared the same as those used in clinical trials. *In vitro* digestion was carried out using dissolution experiments according to Pharmacopoeia official methods (Bio-Dis reciprocating cylinder apparatus 3, Agilent Technologies, Santa Clara, CA, USA) [26]. The temperature of the dissolution media was maintained at 37 ± 0.5 °C. The simulated gastric fluid (SGF) was prepared at pH 1.2 with 0.034 M NaCl. Pepsin was added freshly by vortexing in SGF several times over a period of 5 min. The dissolution experiments were performed at a reciprocating rate of 20 dips per minute (dpm) using mesh screens of 405 µm mesh size. The dissolution outer tubes were filled with 78 mL of SGF, and protein snacks (10 g for Bev-3.0, Bev-7.0 and Gel-Acid; 6.67 g for Gel-Heated together with 3.33 g water) were placed in the inner tube. The ratio of pepsin to whey protein isolate (WPI) was 1:25 on a weight basis. Liquid samples (2 mL) were taken manually at time interval of 10, 20, 30, 60, 90, and 120 min and replenished with 2 mL of fresh SGF. Gel samples were taken according to its remaining weight and broken by sonication using a probe sonicator. Sodium chloride (1 N and 0.1 N) was added to samples to adjust pH to 7.5 to inactivate the enzymes.

Sodium dodecyl sulfate-polyacrylamide gel electrophoresis (SDS-PAGE) was carried out to monitor the degradation of whey protein using a modification of Laemmli method. Samples were solubilized in Laemmli sample buffer (Bio-Rad Laboratories, Hercules, CA, USA) containing 5% β-mercaptoethanol and heated at 95 °C for 5 min, and then loaded (10 µL) onto the polyacrylamide gel (15% acrylamide for resolving gel and 4% acrylamide for stacking gel). The gel was run in a mini Protein II electrophoresis system (Bio-Rad Laboratories) using an electrode stock buffer at a voltage of 200 V and 120 V. The gels were stained with Coomassie brilliant blue R250 in an acetic acid:methanol:H_2_O staining solution (1:4:5 by volume), and destained in an acetic acid:methanol:H_2_O solution (1:4:5 by volume). Unstained molecular weight marker comprising a mixture of protein ranging in size from 5 to 250 kDa was used (PageRuler unstained broad range protein ladder: Thermo Scientific, Rockford, IL, USA). Imaging was accomplished with AlphaImager system (Alpha Innotech Corporation, Santa Clara, CA, USA).

## 3. Results

### 3.1. Appetite

**Figure 1:**
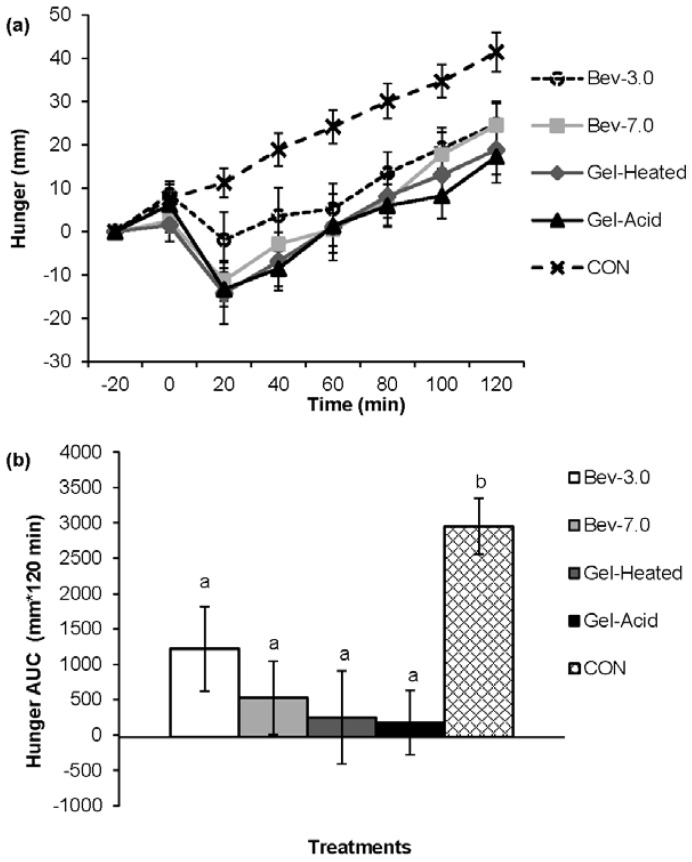
Perceived hunger across time for each treatment (**a**, line graph) and the 2-h net incremental area under the curve (AUC) for the different treatments (**b**, bar graph). Time 0 is when the snack was consumed. Different letters denote significance (*p* < 0.05) between treatments. Snacks: 0 kcal water (CON) or 96 kcal whey protein snacks as beverages with a pH of either 3.0 (Bev-3.0) or 7.0 (Bev-7.0) or gels as acid (Gel-Acid) or heated (Gel-Heated).

**Figure 2:**
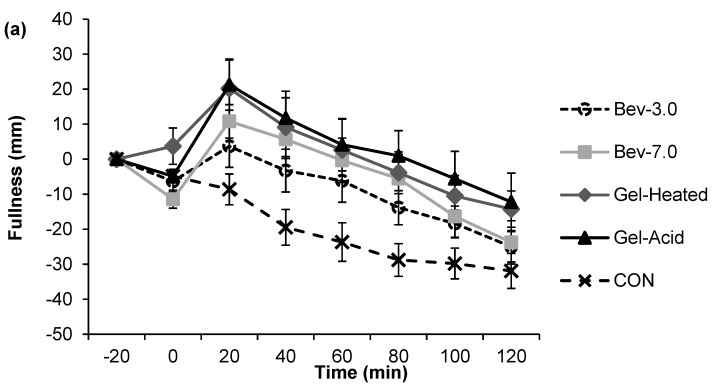

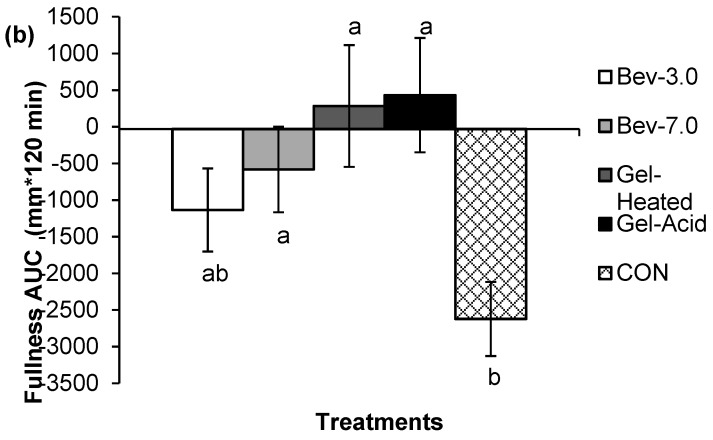
Perceived fullness across time for each treatment (**a**, line graph) and the 2-h net incremental area under the curve (AUC) for the different treatments (**b**, bar graph). Time 0 is when the snack was consumed. Different letters denote significance (*p* < 0.05) between treatments. Snacks: 0 kcal water (CON) or 96 kcal whey protein snacks as beverages with a pH of either 3.0 (Bev-3.0) or 7.0 (Bev-7.0) or gels as acid (Gel-Acid) or heated (Gel-Heated).

**Figure 3:**
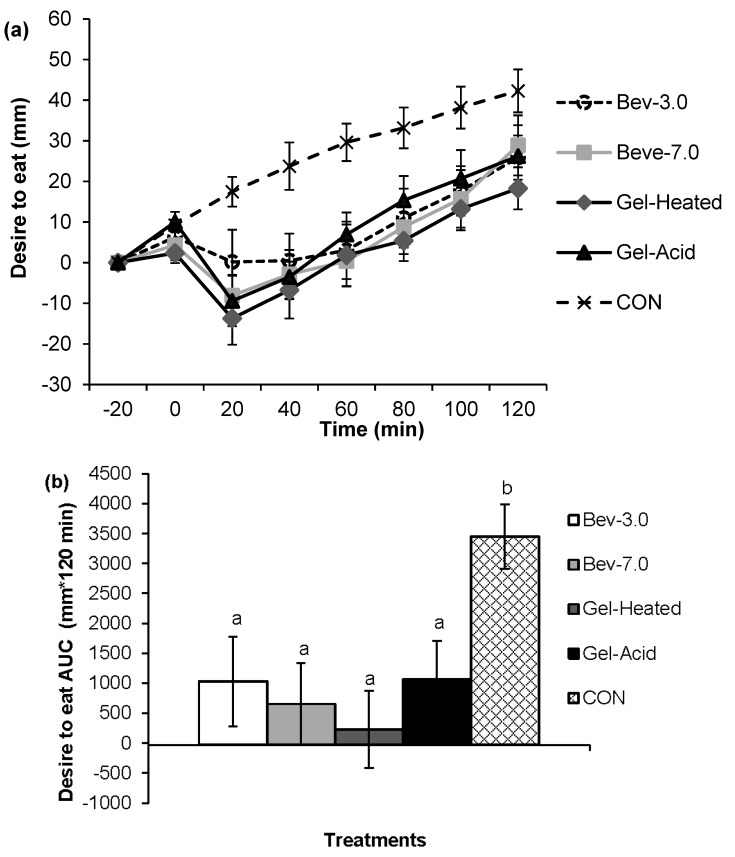
Perceived desire to eat across time for each treatment (**a**, line graph) and the 2-h net incremental area under the curve (AUC) for the different treatments (**b**, bar graph). Time 0 is when the snack was consumed. Different letters denote significance (*p* < 0.05) between treatments. Snacks: 0 kcal water (CON) or 96 kcal whey protein snacks as beverages with a pH of either 3.0 (Bev-3.0) or 7.0 (Bev-7.0) or gels as acid (Gel-Acid) or heated (Gel-Heated).

**Figure 4:**
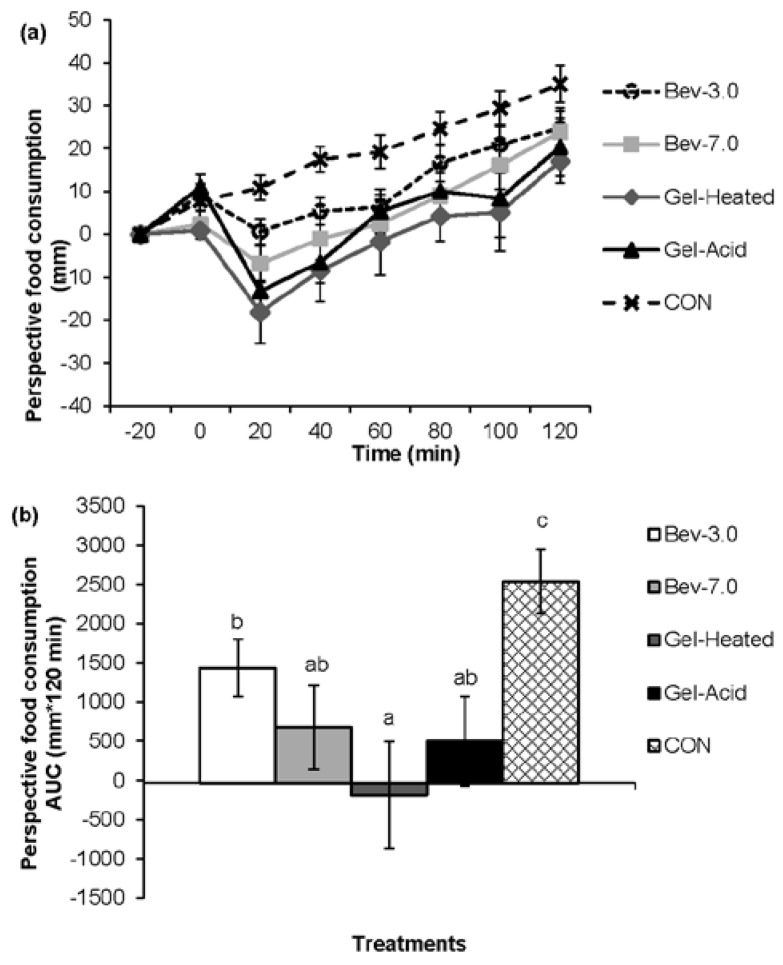
Perceived prospective food consumption across time for each treatment (**a**, line graph) and the 2-h net incremental area under the curve (AUC) for the different treatments (**b**, bar graph). Time 0 is when the snack was consumed. Different letters denote significance (*p* < 0.05) between treatments. Snacks: 0 kcal water (CON) or 96 kcal whey protein snacks as beverages with a pH of either 3.0 (Bev-3.0) or 7.0 (Bev-7.0) or gels as acid (Gel-Acid) or heated (Gel-Heated).

Figure 1, Figure 2, Figure 3 and Figure 4 depict the appetite responses throughout the 2-h post-snack period for the study treatments. The line graphs (a) illustrate the responses completed every 20 min throughout each of the testing days; while the bar graphs (b) depicts the AUC analyses over the post-snack time period.

Post-snack hunger, desire to eat, and prospective food consumption were lower following the mid-morning beverages and gels *vs.* CON (all, *p* < 0.05), and post-snack fullness was greater following the mid-morning snacks (except for the Bev-3.0) *vs.* CON (all, *p* < 0.05). The Gel-Heated treatment led to lower prospective food consumption (AUC: −173.46 ± 653.59) *vs.* Bev-3.0 (AUC: 1322.31 ± 369.75; *p* < 0.05); however, no other differences were detected.

### 3.2. Energy Intake

**Figure 5:**
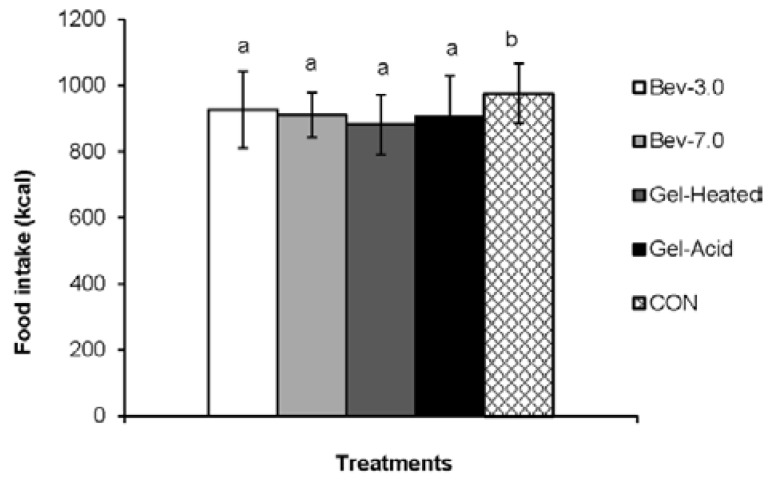
Mean energy intake at the ad libitum lunch for each snack. Different letters denote significance (*p* < 0.05) between treatments. Snacks: 0 kcal water (CON) or 96 kcal whey protein snacks as beverages with a pH of either 3.0 (Bev-3.0) or 7.0 (Bev-7.0) or gels as acid (Gel-Acid) or heated (Gel-Heated).

As shown in Figure 5, the participants consumed 975 ± 90 kcal during the *ad libitum* lunch following the CON, 830 ± 117 kcal with Bev-3.0, 815 ± 67 kcal with Bev-7.0, 785 ± 91 kcal with Gel-Heated, and 809 ± 123 kcal following Gel-Acid. All snacks led to lower energy consumed at the lunch meal *vs.* CON (all, *p* < 0.05). When, assessing dietary compensation of the 96 kcal snack, the consumption of all snacks still resulted in lower energy consumed at lunch *vs.* CON (all, *p* < 0.05). However, no difference in energy content (kcal or dietary compensation) was detected between protein snacks.

## 4. Discussion

We demonstrated that the consumption of a 96 kcal mid-morning whey protein snacks containing 24 g of whey protein improved appetite throughout the morning compared to a 0 kcal water drink and led to reductions in energy intake at the subsequent lunch meal. However, when comparing these effects between the whey protein treatments, food form and structure had little to no impact in healthy adults. These results suggest that whey protein, either as a beverage or a gel snack, has beneficial effects on appetite control and energy intake and may be used as equivalents for weight management.

**Figure 6:**
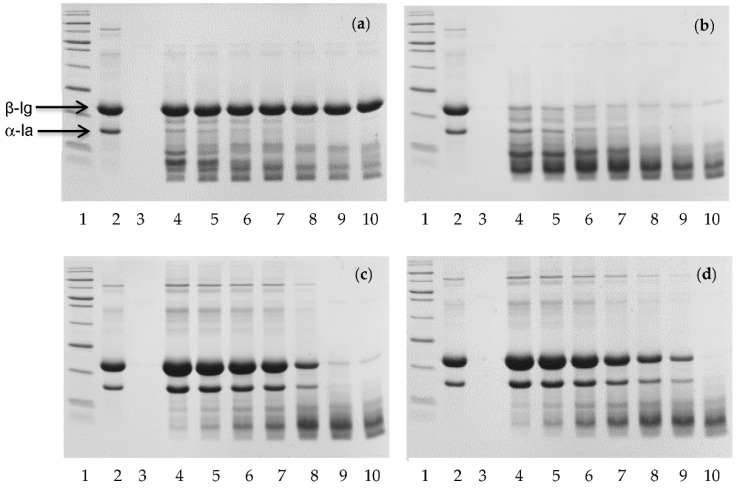
Sodium dodecyl sulfate-polyacrylamide gel electrophoresis (lane 1, maker; lane 2, whey protein isolate (WPI); lane 3, pepsin; lane 4–10, samples being digested for 5, 10, 20, 30, 60, 90, and 120 min) of *in vitro* digestion of whey protein samples: (**a**) protein liquid at pH 3.0; (**b**) protein liquid at pH 7.0; (**c**) heated protein gel at pH 7.0; (**d**) acid protein gel at pH 5.0.

Although *in vitro* digestion models may not truly represent *in vivo* digestion, they offer a rapid approach to determine the structural changes of protein that occur under simulated GI conditions [27]. In this study, *in vitro* digestion of whey protein snacks was performed using the reciprocating cylinder dissolution apparatus which is commonly used to simulate digestion in drug release studies. Electrophoresis was used to monitor the degradation of protein (Figure 6). The β-lactoglobulin monomers of Bev-3.0 were very resistant to gastric digestion, probably due to the limited unfolding and aggregation during heating, hence, only a small amount of peptides were detected in the early stage of digestion. Bev-7.0 showed much faster degradation rate than Bev-3.0 with β-lactoglobulin monomers being digested within 10 min, and much denser peptide bands were detected. Gel-Heated and Gel-Acid had very similar digestion pattern with all monomers being completely digested at 120 min, though heated gel appeared to be digested slightly faster than acid gel. As mentioned above, Gel-Acid at pH 3 was prepared by the addition of GDL to heated protein solution at pH 7. The slowly-released acid from GDL induced gel formation. Thus, the building blocks of the Gel-Acid were heated protein aggregates similar to Bev-7.0. Since the Bev-3.0 was heated at acidic pH, its protein components remained mostly in their monomeric forms unlike the other three samples. The information obtained from the *in vitro* simulation suggests that the susceptibility of protein in GI tract were largely influenced by the protein in different form.

Although *in vitro* simulation showed distinct difference in digestion pattern between Bev-3.0 and Bev-7.0, no significant difference was detected on appetite and satiety response. However, it appears that Bev-3.0 evolved slightly higher response of hunger than Bev-7.0, especially in the first 40 min (Figure 1a). This may be explained by the different digestion patterns of the two beverages in the gastrointestinal tract. It has been reported that mechanisms that may contribute to protein-induced satiety involve increases in concentrations of satiety hormones, concentrations of amino acids, energy expenditure, and the process of gluconeogenesis [28]. The protein in Bev-3.0 was very resistant to hydrolysis, while the protein in Bev-7.0 was much more susceptible to digestion (as shown by *in vitro* result), hence, the peptides and amino acids could have been generated much faster for protein in Bev-7.0, which might elevate blood amino acid concentration and stimulate satiety hormone release, leading to greater reduction in hunger. However, when protein in Bev-3.0 reaches to the small intestine, it was also quickly degraded into small peptides and amino acids, hence, the hunger response in the later stage was similar between the two snacks.

The effect of food form on appetite control has recently received much attention due to the difference observed between liquid foods and solid foods. There is strong evidence that liquid foods elicit weaker appetite and satiety responses than solid foods [9,10,11,12]. Furthermore, increase in fullness occurs more rapidly and exists for a longer time period following solid food consumption compared to liquid food consumption [29]. In this work, no significant difference of appetite and satiety response was observed between beverage and gel. There are several reasons that may account for this phenomenon. The large differences in the volume and energy density of liquids and solids have been reported as one of the reasons that solids evoked stronger satiety [10]. Meals with larger volume and lower energy density have faster gastric emptying rate and evoke weaker satiety signals [10]. In the present study, whey protein snack was served in nutritionally identical forms as either beverage or gel. Gel-acid was simply formed by adding GDL to Bev-7.0, so the total volume served was the same as that of the beverages. The serving size of Gel-Heated (formed at 15% protein) was 200 g but additional 100 g of water was also served to control the volume. It is possible that the lack of significant difference among Bev-7.0, Gel-Acid, and Gel-Heated was due to their similarity in digestion pattern and similar volume consumed. The other reason could be due to the softer texture of the gel compared to solid foods in other studies. It has been reported that whether consumed with a meal or alone as a snack, semi-solid food elicited weaker appetitive response than solid food [30]. The Gel-heated prepared in this study was weaker compared to normal solid foods and its texture was similar to gummy bear though not as elastic. The Gel-Acid was much weaker, thus the two gels did not require a lot of chewing. The mastication process of solid foods has been reported to contribute to satiety [31].

Whey protein preloads showed much lowered energy intake during lunch compared to water preloads; however, no significant difference was observed between protein snacks. Numerous reports have demonstrated that consumption of whey protein reduces *ad libitum* food intake at the test meal. Pal *et al.* (2010) [32] reported that energy intake following water preload was higher than following whey protein preloads. Akhavan *et al.* (2010) [33] reported that the dose of whey protein required to suppress food intake when consumed 30 min before the meal ranged from 20 to 40 g with a sample size of 16, while only 10 g protein was needed with a sample size of 40. In the present study, 24 g of whey protein was sufficient to suppress food intake 2 h after preloads, despite the form of whey protein. There are few conclusive studies investigating the effect of physical form of protein preload on subsequent food intake. It was reported that solid-meal replacement product had lower hunger, desire to eat, insulin, and ghrelin responses compared to the liquid version, but the effect on energy intake was unclear [29]. Although other studies reported no difference in energy intakes between a beverage and a solid food, accumulating evidence suggested that liquid carbohydrates generally reduce food intake more than solid forms [9,34,35]. Future studies examining the effect of physical form of different protein on satiety and subsequent food intake are needed.

One of the limitations of the current study is that we did not monitor the change of postprandial glycemic response and appetite-related hormones after whey protein preload, which may have provided greater insight into the effect of physical form of protein on appetite suppression. It has been reported that consumption of whey protein lowered blood glucose response and fasten postprandial gut hormones release, which contribute to the satiety and food intake regulation [6,28,33,36]. Another limitation of this study is that whey protein gels used as protein solid form are not actual foods that subjects consume in their daily life. This protocol was designed to minimize the effect of other food components on satiety, by matching protein snack for energy, weight, and volume. If using actual protein gel food, such as yogurt or mousse, other food components such as carbohydrate will be incorporated, hence, we cannot rule out that the outcome is only due to the effect of protein. In addition, the gels and beverages were not of the same flavor. Although sensory attributes did not act as covariates on all the study outcomes, it is unclear whether the unconventional protein gel samples would influence the rating of satiety and energy intake. Furthermore, compared to common solid foods, semi-solid protein gel is weaker and easier to be degraded, which might be one of the reasons that no difference was observed between beverage and gel snacks.

## 5. Conclusions

In conclusion, no significant difference of appetite, satiety response, and energy intake was observed between beverage and gel protein snacks. Food form or physico-chemical properties elicit little to no effect on these outcomes. However, compared to water, the consumption of a 24 g whey protein, mid-morning snack, as a beverage or a gel, improves appetite control and reduces subsequent energy intake in healthy adults. Further work is required to understand the effects of physical form and protein structure on satiety and energy intake after consumption of whey protein. Furthermore, factors in determining appetite and food intake other than the physical state of protein need to be identified.
